# Supplementary material for: Tunable electronic structure and magnetic anisotropy in bilayer ferromagnetic semiconductor Cr2Ge2Te6
Source: Sci Rep. 2021 Feb 2;11:2744. doi: 10.1038/s41598-021-82394-y (PMC7854638; doi:10.1038/s41598-021-82394-y)
Supplement: Supplementary file 1 — Supplementary Information [file 41598_2021_82394_MOESM1_ESM.pdf]

Supplemental materials

Tunable electronic structure and magnetic anisotropy in bilayer ferromagnetic semiconductor $Cr_2Ge_2Te_6$

Wen-ning Ren[1,2], Kui-juan Jin[1,2,3,*], Jie-su Wang[1], Chen Ge[1,2], Er-Jia Guo[1,2], Cheng Ma[1,2], Can Wang[1,2,3], Xiulai Xu[1,2,3]

[1]*Beijing National Laboratory for Condensed Matter Physics, Institute of Physics, Chinese Academy of Sciences, Beijing 100190, China*

[2]*School of Physical Sciences, University of Chinese Academy of Sciences, Beijing 100049, China*

[3]*Songshan Lake Materials Laboratory, Dongguan 523808, China*

[*]Correspondence and requests for materials should be addressed to Kuijuan Jin: kjjin@iphy.ac.cn

The checking calculations of U_{eff} parameters for the bilayer $Cr_2Ge_2Te_6$ are conducted and the results are shown in Figs. S1, S2, and S3. The lattice constants, magnetic property, and electronic structures of U_{eff} =1.7 eV are in line with previous calculated and experimental results in Ref. [1-3]. As shown in Fig. S1(a), we obtain the lattice parameters a=b=6.838 Å, with a difference of 0.15% with respect to the experimental measurements reported by V. Carteaux *et al* [2]. Figure. S1(b) shows that the out-of-plane anisotropy energy is obtained while the ferromagnetic ground state maintains by using U_{eff} =1.7 eV. As shown in Fig. S2, the spin polarized characteristics of the electron at the conduction minimum band (CBM) and valence maximum band (VBM) are affected by different U_{eff}. Herein, by using U_{eff} =1.7 eV, the calculated band structures indicate that both the CBM and VBM are with purely spin-up state, and the calculated band gap is also in good agreement with previous theoretical value [1], as shown in Fig. S3.

Figure S1: The effective on-site Coulomb energy U_{eff} dependence of (a) lattice constant a, (b) magnetic anisotropy energy (MAE) and magnetic ground state. The magenta horizontal dashed lines are located at the previous experimental values [2], just for comparison. The gray horizontal dashed lines denote the boundary of spin polarization or magnetic ground state. The arrows point to the axises for each curve in the corresponding color.

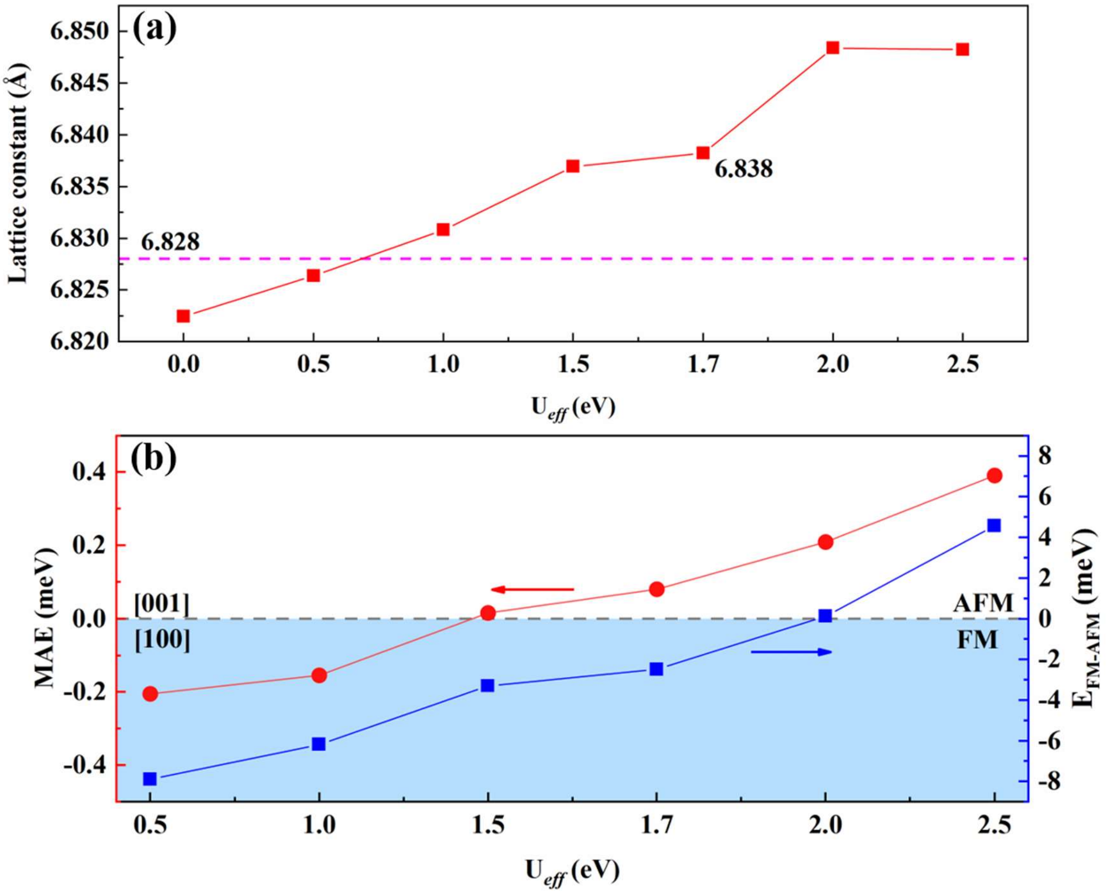

Figure S2: The effective on-site Coulomb energy U_{eff} dependence of band structures. The gray horizontal dashed lines denote the Fermi level. To achieve the visualizations of band gaps, the CBM and VBM are connected by black arrows.

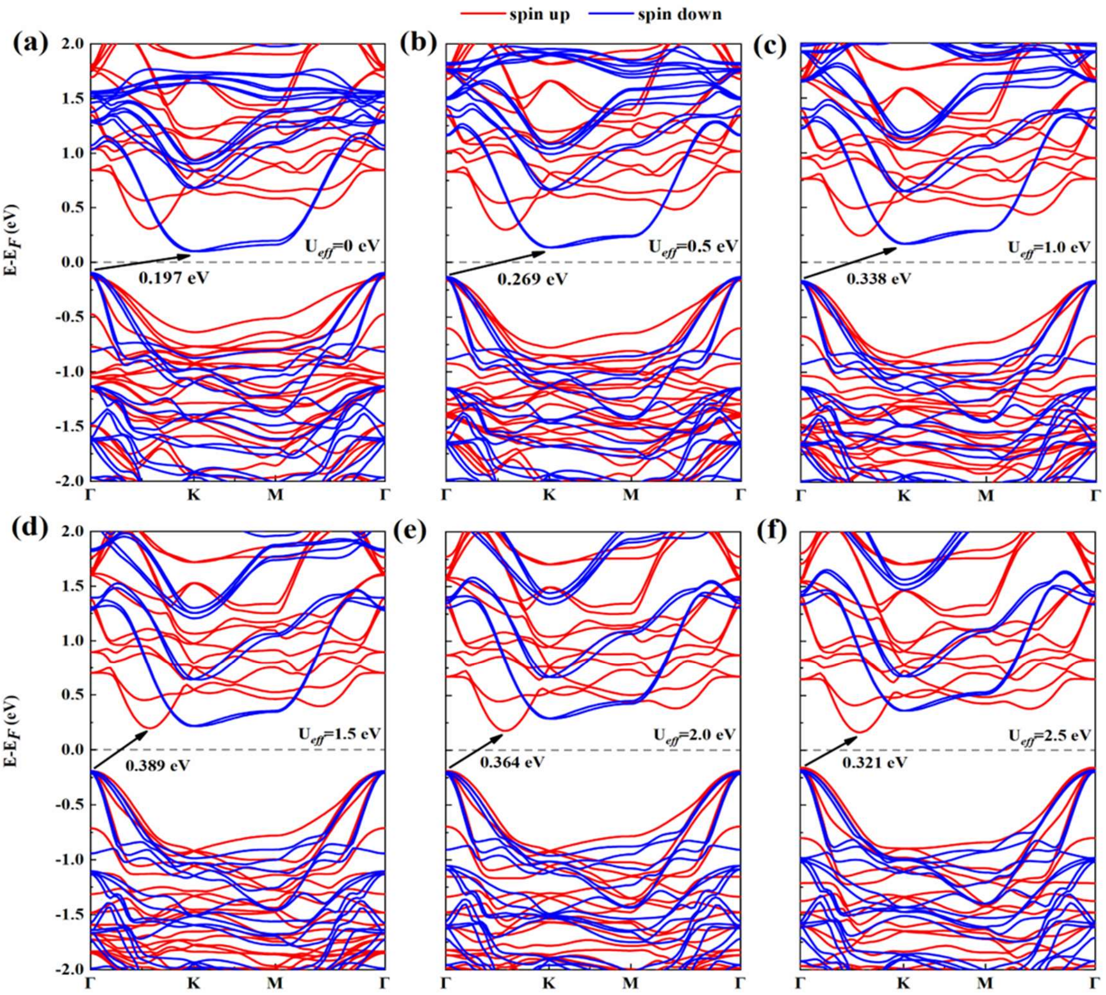

Figure S3: The effective on-site Coulomb energy U_{eff} dependence of band gap. The cyan horizontal dashed lines denote the previous calculated value [1].

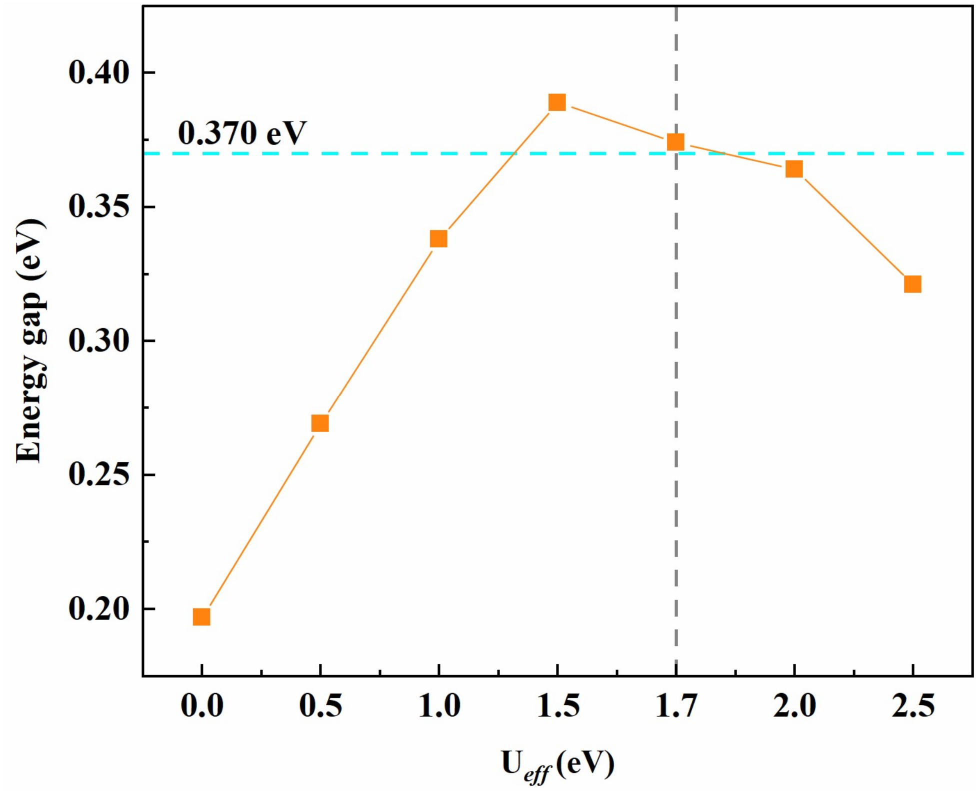

References

1. Fang Y. M., Wu S. Q., Zhu Z.-Z. & Guo G.-Y., Large magneto-optical effects and magnetic anisotropy energy in two-dimensional $Cr_2Ge_2Te_6$. *Phys. Rev. B* **98** 125416 (2018).
2. Carteaux, V., Brunet, D., Ouvrard, G. & Andre, G. CRYSTALLOGRAPHIC, MAGNETIC AND ELECTRONIC-STRUCTURES OF A NEW LAYERED FERROMAGNETIC COMPOUND $CR_2GE_2TE_6$. *Journal of Physics-Condensed Matter* **7**, 69-87, (1995).
3. Gong C. *et al*. Discovery of intrinsic ferromagnetism in two-dimensional van der Waals crystals. *Nature* (London) **546**, 265 (2017).
